# Approaches and methods to study wildlife cancer

**DOI:** 10.1111/1365-2656.14144

**Published:** 2024-08-27

**Authors:** Mathieu Giraudeau, Orsolya Vincze, Sophie M. Dupont, Tuul Sepp, Ciara Baines, Jean-Francois Lemaitre, Karin Lemberger, Sophie Gentès, Amy Boddy, Antoine M. Dujon, Georgina Bramwell, Valerie Harris, Beata Ujvari, Catherine Alix-Panabières, Stephane Lair, David Sayag, Dalia A. Conde, Fernando Colchero, Tara M. Harrison, Samuel Pavard, Benjamin Padilla-Morales, Damien Chevallier, Rodrigo Hamede, Benjamin Roche, Tamas Malkocs, Athena C. Aktipis, Carlo Maley, James DeGregori, Guillaume Le Loc’h, Frédéric Thomas

**Affiliations:** 1 Littoral Environnement et Sociétés (LIENSs), UMR 7266 CNRS-La Rochelle Université, La Rochelle, France; 2 ImmunoConcEpT, CNRS UMR 5164, University of Bordeaux, Bordeaux, France; 3 Hungarian Department of Biology and Ecology, Evolutionary Ecology Group, Babeş-Bolyai University, Cluj-Napoca, Romania; 4 HUN-REN-DE Conservation Biology Research Group, Debrecen, Hungary; 5 Laboratoire de Biologie des ORganismes et Ecosystèmes Aquatiques (BOREA), FRE 2030, Muséum National d’Histoire Naturelle, CNRS, IRD, Sorbonne Université, Université de Caen Normandie, Université des Antilles, Paris, France; 6 Institute of Ecology and Earth Sciences, University of Tartu, Tartu, Estonia; 7 Department of Biological Sciences, University of Leeds, Leeds, United Kingdom; 8 Laboratoire de Biométrie et Biologie Évolutive, CNRS, UMR5558, Université Lyon 1, Villeurbanne, France; 9 Vet Diagnostics, Lyon, France; 10 Department of Anthropology, University of California Santa Barbara, Santa Barbara, California, USA; 11 School of Life and Environmental Sciences, Deakin University, Waurn Ponds, Victoria, Australia; 12 CREEC/CANECEV, MIVEGEC, Unité Mixte de Recherches, IRD 224–CNRS5290–Université de Montpellier, Montpellier, France; 13 Arizona Cancer Evolution Center, Biodesign Institute, Arizona State University, Tempe, Arizona, USA; 14 Centre de Recherches Ecologiques et Evolutives sur le Cancer, Montpellier, France;; 15 Laboratory of Rare Human Circulating Cells (LCCRH), University Medical Centre of Montpellier, Montpellier, France; 16 Faculté de médecine vétérinaire, Canadian Wildlife Health Cooperative/Centre québécois sur la santé des animaux sauvages, Université de Montréal, Saint-Hyacinthe, Quebec, Canada; 17 ONCOnseil—Unité d’expertise en oncologie vétérinaire, Toulouse, France; 18 Department of Biology, University of Southern Denmark, Odense M, Denmark; 19 Interdisciplinary Centre on Population Dynamics, University of Southern Denmark, Odense M, Denmark; 20 Department of Primate Behavior and Evolution, Max Planck Institute for Evolutionary Anthropology, Leipzig, Germany; 21 Department of Mathematics and Computer Sciences, University of Southern Denmark, Odense M, Denmark; 22 Department of Clinical Sciences, College of Veterinary Medicine, North Carolina State University, Raleigh, North Carolina, USA; 23 Unité Eco-Anthropologie (EA), Muséum National d’Histoire Naturelle, CNRS 7206, Université Paris Cité, Paris, France; 24 Department of Biology and Biochemistry, Milner Centre for Evolution, University of Bath, Bath, UK; 25 School of Natural Sciences, University of Tasmania, Hobart, Tasmania, Australia; 26 Centre de Recherche en Écologie et Évolution de la Santé (CREES), Montpellier, France; 27 Departamento de Etología, Fauna Silvestre y Animales de Laboratorio, Facultad de Medicina Veterinaria y Zootecnia, Universidad Nacional Autónoma de México (UNAM), Ciudad de México, Mexico; 28 Univ Brest, CNRS, IRD, Ifremer, LEMAR, IUEM, Plouzane, France; 29 Department of Psychology, Arizona State University, Tempe, Arizona, USA; 30 Department of Biochemistry and Molecular Genetics, University of Colorado School of Medicine, Aurora, Colorado, USA; 31 École Nationale Vétérinaire de Toulouse, Toulouse, France

**Keywords:** cancer, cancer diagnostic, disease ecology, One Health, wildlife disease

## Abstract

1. The last few years have seen a surge of interest from field ecologists and evolutionary biologists to study neoplasia and cancer in wildlife. This contributes to the One Health Approach, which investigates health issues at the intersection of people, wild and domestic animals, together with their changing environments. Nonetheless, the emerging field of wildlife cancer is currently constrained by methodological limitations in detecting cancer using non-invasive sampling. In addition, the suspected differential susceptibility and resistance of species to cancer often make the choice of a unique model species difficult for field biologists.
2. Here, we provide an overview of the importance of pursuing the study of cancer in non-model organisms and we review the currently available methods to detect, measure and quantify cancer in the wild, as well as the methodological limitations to be overcome to develop novel approaches inspired by diagnostic techniques used in human medicine.
3. The methodology we propose here will help understand and hopefully fight this major disease by generating general knowledge about cancer, variation in its rates, tumour-suppressor mechanisms across species as well as its link to life history and physiological characters. Moreover, this is expected to provide key information about cancer in wildlife, which is a top priority due to the accelerated anthropogenic change in the past decades that might favour cancer progression in wild populations.

## 1 | INTRODUCTION

Tumours, also known as neoplasms, are abnormal masses of tissue that result from abnormal cell proliferation, where cells divide uncontrollably and fail to undergo cell death, when they are supposed to. Tumours can be classified as benign or malignant. Benign tumours are non-cancerous growths, that do not invade organs and tissues beyond the tissue of their primary occurrence and they are rarely lethal to the host. Malignant tumours, also known as cancer, on the other hand, possess the ability to spread to distant organs and tissues within the host (known as metastasis) and sooner or later they start to interfere with the normal functioning of the hosts’ organs and tissues (Box 1).

Tumours affect the majority, if not all, metazoans (Aktipis et al., 2015; Thomas et al., 2017). Even the once presumed cancer-resistant naked mole rat (*Heterocephalus glaber*) has been revealed to exhibit cancer at exceptionally low rates (Delaney et al., 2016). In contrast, certain species, such as the Tasmanian devil (*Sarcophilus harrisii*, McCallum et al., 2009), the green sea turtle (*Chelonia mydas*, Chaloupka et al., 2009) or the beluga whale (*Delphinapterus leucas*, Martineau et al., 2002), exhibit markedly high prevalence of tumours, posing a threat to the conservation of these species, in some cases even pushing these taxa to the brink of extinction. Beyond this concerning observation, neoplasia rates are predicted to increase in wild populations in our rapidly changing world, with most species being now impacted by human activities (Giraudeau et al., 2018; Sepp et al., 2019). For instance, the contamination of aquatic environments with carcinogenic pollutants has been shown to increase neoplasia risk in freshwater (Black et al., 1982), as well as marine fishes (Lerebours et al., 2014). Moreover, the extensive release of pollutants globally is anticipated to extend this pattern and impact a broader range of wild organisms and habitats in the near future (Giraudeau et al., 2018; McAloose & Newton, 2009).

Cancer, marked by substantial mortality rates in humans, has been the focus of intense scientific scrutiny, particularly in recent decades. In contrast, reports of neoplasia in wildlife have only recently begun to emerge from veterinarians and wildlife health centres (Pewsner et al., 2017), and these cases often lacked subsequent follow-up or surveillance, primarily due to financial or infrastructural constraints. Investigations into wildlife neoplasia have then been limited to a handful of species, focusing mainly on transmissible cancers or cancers associated with oncogenic viruses (Dujon, Schofield, et al., 2020). This might appear surprising given the suspected role of the oncobiota (i.e., community of cancerous cells, from precancerous lesions to metastatic cancers) in animal ecology and ecosystem functioning (Vittecoq et al., 2013). This lack of interest and investment from the scientific community and funding bodies in wildlife cancer has been driven by the formerly widely claimed scarcity of cancers (especially metastatic ones) in nature (e.g., Munson & Moresco, 2007). On the contrary, oncogenic phenomena are now recognized to be highly prevalent in wildlife (Madsen et al., 2017; Vincze et al., 2022). Nevertheless, detecting and confirming tumours in wildlife remain challenging, with carcasses rarely being recovered and histopathological examinations (if any) commonly performed on decomposing carcasses presenting high levels of autolysis (McAloose & Newton, 2009).

From a biological point of view, cancer can be considered as a long process starting early in life with the appearance of driver mutations and precancerous lesions, potentially impacting individuals long before the end of their reproductive period. In fact, wild organisms suffering from early stages of cancer are predicted to be more susceptible to premature death by predation or parasitism (Vittecoq et al., 2013), thereby removing cancerous individuals from the population prior to the development of metastatic cancers. In this context, there is a growing urgency to determine the proximate causes of cancer; evaluate its ecological, evolutionary and demographic implications in wild populations; and consider not only the late stages of cancer, but also the entire oncobiota at the individual, population and species levels. Leveraging evolutionary perspectives on this disease holds promise for shaping conservation policies aimed at safeguarding wild populations, a crucial endeavour in the face of the ongoing extinction crisis driven by anthropogenic threats such as habitat loss, pollution, overexploitation and climate change (Ceballos et al., 2017).

**FIGURE 1:**
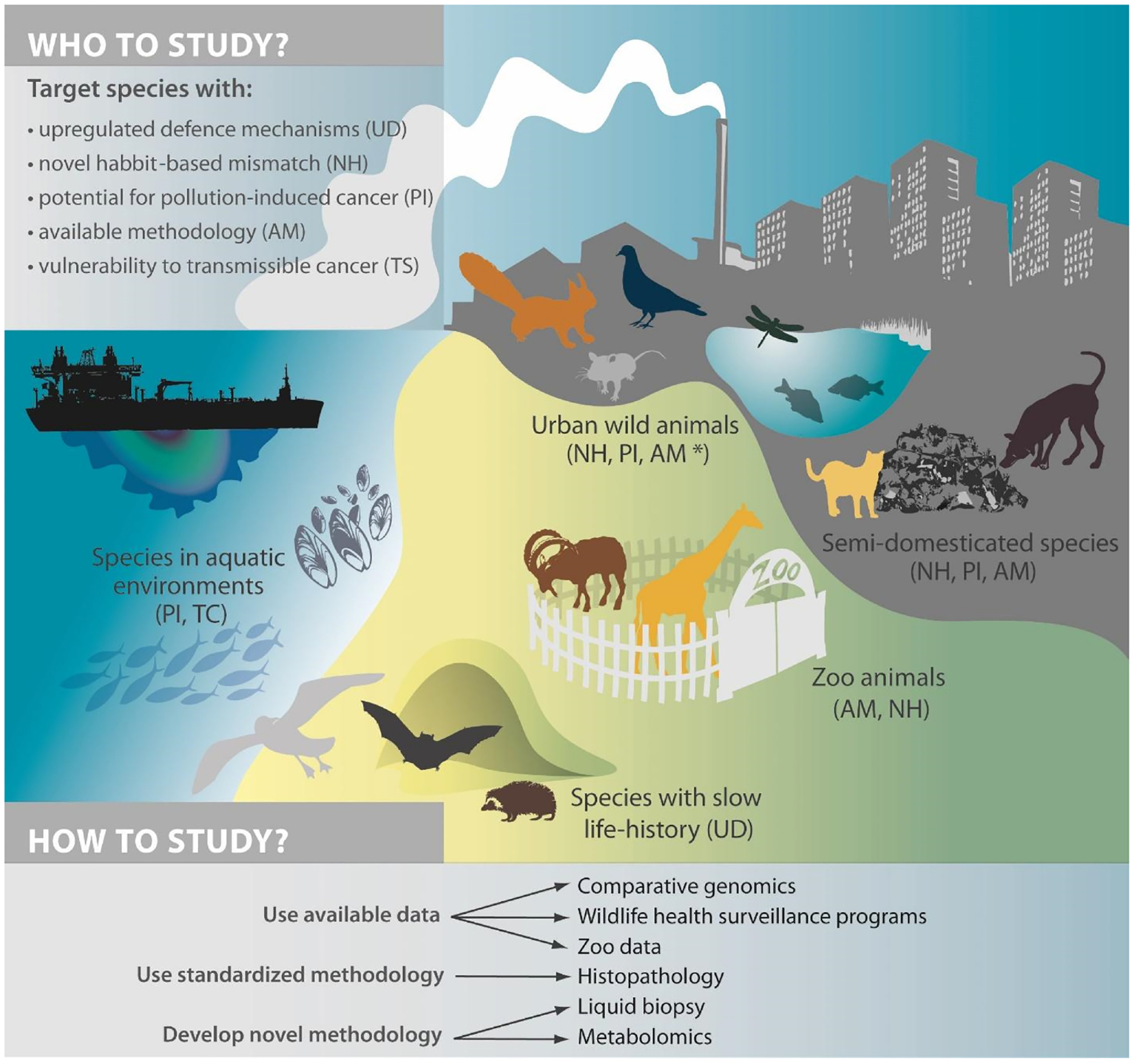
Summary of species of interest and methods to study wildlife cancer (*available methodology if focusing on mice and rats).

The last few years have seen a surge of interest from field ecologists and evolutionary biologists to study wildlife cancer in the context of global change (Giraudeau et al., 2018, 2020; Meitern et al., 2020; Pesavento et al., 2018; Sepp et al., 2018), thus contributing to the One Health Approach (Text Box 2). This integrated and unified approach investigates health issues at the intersection of people, wild and domestic animals, together with their changing environments. However, the emerging field of wildlife cancer is currently constrained by methodological limitations in detecting early-stage cancers using non-invasive sampling. In addition, the suspected differential susceptibility and resistance of species to cancer make the choice of a unique wild model species difficult for field biologists. We propose a theoretical framework to study cancer in wildlife and highlight future avenues in the identification of efficient tumour-suppressor mechanisms. We also provide a review of currently available methods to detect, measure and quantify animal cancer in the wild, as well as the methodological limitations that need to be overcome to develop novel approaches inspired by diagnostic techniques used in human medicine (Figure 1).

Importantly, for the purpose of this paper, we considered that differential levels of intrinsic anticancer mechanisms between species and/or exposure to diverse oncogenic factors should make animals more or less prone to all or most of the different types of neoplasms. Of note, different types of neoplasia have already been documented in animals (including wildlife), such as hereditary neoplasms, spontaneous neoplasms (Vincze et al., 2022), infectious (generally virally induced) neoplasms (Aguirre & Spraker, 1996), contagious neoplasms (e.g., canine transmissible venereal tumour [CTVT], devil facial tumour disease [DFTD], bivalve transmissible neoplasia, Dujon, Schofield, et al., 2020), as well as neoplasia that occurs secondary to chronic inflammation, or following exposure to oncogenic compounds (such as radioactive materials or toxins for instance, Baines et al., 2021). Thus, when studying wildlife cancer, each model system, population and neoplasm type has its own characteristics and all the methodologies and theoretical background provided by this paper cannot be applied to all of them. Instead, ecologists and evolutionary biologists interested in this topic should target the methodology offered by this paper in relation to the scientific questions addressed by their project and the system they are planning to use.

## 2 | STUDY SPECIES FOR WILDLIFE CANCER

In this section, we propose potential species to target when studying cancer in wildlife. These species are suggested based on specific research questions that can be addressed using them, or based on their low predicted or measured cancer prevalence, as well as the availability of techniques that can be used for quantifying cancer progression.

### 2.1 | Embracing the diversity of life-history strategies

Laboratory rodents are popular models in biomedical research, but extrapolating findings from laboratory-based rodent studies to other organisms is far from straightforward (Perlman, 2016), especially when it comes to cancer research (Anisimov et al., 2005). In fact, laboratory rodents are notorious for having accumulated an unusually large number of derived traits and mutations that differentiate them from other mammals (Miller et al., 2002). Among the diversity of life history strategies observed across vertebrates, most rodents are located at the fast end of the slow–fast continuum, meaning that they display a covariation of short biological times (e.g., fast growth period, short gestation time and reduced lifespan) (Stearns, 1983). This slow–fast continuum (structured by a trade-off between fecundity and survival) constitutes the main axis of life history strategies in mammals (Healy et al., 2019), and explains half of the variation in life history strategies across vertebrates (Gaillard et al., 2016). Moreover, many life history traits, including lifespan and ageing parameters (e.g., rate, onset), covary with body size following an allometric relationship (Peters, 1983). Therefore, focusing on species living longer lives than expected for their body size, their position along the slow–fast continuum, or both, might be a promising strategy to identify anticancer mechanisms that have evolved in specific species or lineages (Vincze et al., 2022). This approach has already been applied among rodents (see Gorbunova et al., 2014, for a focus on the iconic naked mole rat, and the Middle East blind mole rat, *Nannospalax ehrenbergi*), and it could be extended to other taxonomic groups where such species have already been identified (see Wilkinson & Adams, 2019, for the specific case of bats).

Interestingly, specific ecological traits (e.g., tropical habitats) or lifestyles (e.g., hibernation and sociality) have been suggested to be associated with a slowing down of the pace of life and, consequently, with life history traits (and underlying mechanisms) favouring survival over reproduction (Gaillard et al., 2016). For instance, hibernating species show higher annual survival rates than non-hibernating species of a similar size (Turbill et al., 2011). Whether the extended lifespan associated with hibernation has co-evolved with specific physiological features providing a better resistance to cancer is yet to be determined. In any case, species with extreme longevity or those displaying low or negligible actuarial senescence (e.g., Cayuela et al., 2019) may be informative biological models for studying cancer in the wild.

### 2.2 | Species in evolutionary mismatch

Evolutionary theory predicts that, in natural environments, the evolution of suppressive cancer mechanisms is traded against other fitness-related functions (Boddy et al., 2015). Moreover, natural selection adjusts these conflicting demands through time in a way that determines the best host strategy to achieve maximal fitness given environmental conditions (Jacqueline et al., 2017). As a result, metastatic cancer risks, although not eliminated, are often reduced in animals and occur mostly in old age, when selection to maintain efficient cancer defence mechanisms is weakened (DeGregori, 2011).

When environmental conditions change rapidly through time or space, previous equilibria may no longer be optimal for host fitness (i.e., some evolved traits that were initially advantageous could become maladaptive). The temporary period of disequilibrium before the population readapts to the new conditions is called a ‘mismatch’ (Greaves, 2015). Evolutionary mismatches have often been hypothesized to increase cancer risk. For instance, ecological conditions in our modern world are radically altered by human activities, resulting in a mismatch with our inherent genetic architecture, that was shaped by ancestral and very different environmental circumstances (Greaves & Aktipis, 2016). It is now believed that increased susceptibility to several cancers in humans is partly due to mismatches between the altered environment and slowly evolving cancer suppression mechanisms (Greaves & Aktipis, 2016). As suggested by Giraudeau and colleagues, wildlife species are often collateral victims of environmental changes (Giraudeau et al., 2018). Wildlife species are currently believed to experience a higher rate of cancers than in the past due to exposure to modern human-induced mismatches (Baines et al., 2021; Martineau et al., 2002). Recently, Thomas and colleagues also argued that the domestication process, initiated by humans during the Neolithic more than 12,000 years ago, has placed animals (e.g., dogs, chicken and cows) in unprecedented ecological and genetic mismatches in which cancer risks are often exacerbated (Thomas et al., 2020). For instance, the higher incidence of bone cancer in large dogs is at least partially attributed to artificial selection for larger size (Nunney, 2013).

Compared with the vast attention that has been dedicated to exploring the oncogenic consequences of human-induced mismatches, few studies have focused on the mismatches that could result from natural evolutionary changes (i.e., those not directly related to human activity). Because environmental conditions always change through time and/or space, organisms naturally and constantly evolve, changes in phenotype in traits such as size, metabolism, morphology and/or longevity are frequent. Leroi et al. (2003) predicted that the selection of mechanisms to prevent or alleviate fitness costs due to cancer should be especially intense when animals evolve new morphologies or acquire larger bodies and longer lifespans. In accordance with this prediction, large and long-living animal species have been shown to possess additional protections against cancer (e.g., Abegglen et al., 2015). However, this phenomenon is only the successful result of a selection that necessarily takes more or less time to occur, depending on the species’ biology and/or the ecological context (Nunney, 2013).

Species are expected to transiently experience an evolutionary mismatch between their risk of developing cancer and their level of cancer defence during evolution (see menopause as a possible example in humans, Thomas et al., 2019). Evolutionary mismatches can be resolved by the evolution of new, effective cancer defences and/or compensatory life history traits preventing invasive cancer occurrence. When observed during the selection episode, with all things being equal, these species are thus expected to be transiently at a higher risk of cancer compared with species that are more stably adapted to their environment. It might also be expected that the oncogenic consequences for the former will be correlated with evolutionary mismatch intensity, that is being higher when environmental changes are rapid and drastic, such as after an ecological disaster or habitat change, than when changes are slow and gradual. The rate at which additional cancer defences are selected is likely to be influenced by several parameters, including the genetic variability of the species, mutation rate and genetic drift. In addition, depending on the magnitude of the reproductive benefits associated with the acquisition of novel phenotypic traits (e.g., change in life history traits with higher fecundity, higher size-related sexual competitiveness), the net fitness of evolving individuals may be high despite enhanced cancer risks, yielding to antagonistic pleiotropy that would slow down the selection of stronger cancer defences.

To our knowledge, these predictions have not yet been rigorously tested empirically or theoretically, but they appear to offer promising explanations (at least partially) for the differential vulnerabilities of species to cancer (Vincze et al., 2022). We encourage scientists to explore whether species currently displaying the highest rate of cancers in the field also correspond to species experiencing rapid and recent evolutionary changes. We also predict that species that colonize novel habitats should be, at least transiently, exposed to a higher risk of cancer, especially when phenotypic changes favoured in the novel habitat accentuate the evolutionary mismatch between cancer risk and cancer defences (e.g., a larger size and/or longevity, shift in diet or exposure to novel carcinogens). The extent to which successful invasive species correspond to species that intrinsically have a low vulnerability to cancer, and/or rapidly fix efficient cancer defences, also deserves to be explored.

### 2.3 | Examples of evolutionary mismatches that deserve attention in the context of wildlife cancer

Species in an evolutionary mismatch can show a higher susceptibility to cancer because their defence mechanisms do not adjust yet to the current environmental conditions. Here, we provide two examples that could constitute new avenues of research in this emerging field of study.

#### 2.3.1 | Pollution and cancer in aquatic environments

Cancer in aquatic biota occurs across a number of phyla (from molluscs to mammals) and increased pollution has been suggested as a contributing factor (Baines et al., 2021). Aquatic environments are under extensive threat from various pollutants, particularly in areas with high industrial or agricultural activity. A number of these pollutants, particularly polycyclic aromatic hydrocarbons (PAHs), polychlorinated biphenyls (PCBs), heavy metals and a number of pesticides have been classified as carcinogenic or probable carcinogenic to humans by the International Agency for Research on Cancer (IARC, see IARC Monographs).

Links between PAHs (e.g., Benzo[a]pyrene, BaP) and cancer formation have been suggested in a number of species. The brown bullhead (*Ameiurus nebulosus*) shows rates of cancer as high as 41.1% when exposed to PAHs (Baumann & Harshbarger, 1995). Similarly, the sauger (*Sander canadensis*) develops hepatocellular carcinomas and dermal fibromas when exposed to metals from copper-mining activities (Black et al., 1982). High levels of tissue-accumulated cadmium are linked to increased risk of hepatocellular adenoma and hepatocellular carcinoma development in the common dab (*Limanda limanda*) (Lerebours et al., 2014). A number of pesticides have also been suggested as mutagenic and carcinogenic and some have been banned (at least regionally) as a result of their environmental impacts (e.g., the agricultural use of DDT has been banned in most developed countries by the 1980s). However, these chemicals are often persistent and remain present in aquatic environments for decades after initial exposures and may still affect cancer rates in aquatic organisms (Browning et al., 2015).

Understanding the extent to which these pollutants contribute to cancer formation in aquatic species is paramount in guiding policies related to the use and release of these pollutants in the environment. For instance, it would be indispensable to investigate how pollutants affect host physiology in respect to cancer development, as well as the interactions between hosts and oncogenic viruses (Ylitalo et al., 2005). Many chemicals promote cancer by a direct, mutagenic effect, but pollutants often also interfere with the normal functioning of the host’s immune system (reviewed in Kataoka & Kashiwada, 2021), ultimately increasing their susceptibility to infections, such as by oncogenic viruses (Gauthier et al., 1999).

One of the major challenges with understanding how pollution influences cancer risk is that many wild species are subjected to a cocktail of pollutants in natural aquatic environments, making it difficult to discriminate the effect of each individual pollutant from the cocktail effect. For example, cancer prevalence was monitored in European eels (*Anguilla anguilla*) exposed to a diversity of pesticides, PAHs and heavy metals of varying concentrations at three distinct sites in the Camargue National Reserve, France (Oliveira Ribeiro et al., 2005). Two of the sites appeared to have higher concentrations of pesticides and PAHs based on bile samples from the eels. Interestingly, they discovered that liver and spleen neoplasia were more common in the less polluted site (30% of eels) than in the two polluted sites (0% and 17%), suggesting that another, non-measured pollutant may trigger cancer development in this species. Alternatively, non-measured environmental variables may exacerbate the effects of some pollutants, or factors, besides pollutants, might trigger cancer development in eels on the studied site (Oliveira Ribeiro et al., 2005).

Compared with the open ocean, freshwater and coastal systems are often exposed to higher concentrations of contaminants due to their specific hydrodynamics and the terrestrial nature of most pollution sources. However, the ocean floor can act as a major sink for marine pollution, with benthic species potentially being exposed to larger concentrations of pollutants than pelagic species. Many benthic marine species are already used as model organisms for studying the impact of pollution on cancer formation (Baines et al., 2021; Sakurai et al., 2009), and continuing this work will be important in gaining a better understanding of individual and combined pollutant contributions to cancer in aquatic organisms.

#### 2.3.2 | Urbanized wild animals and anthropogenic food

As human impact extends into natural habitats, a potential source of evolutionary mismatch that could contribute to an elevated cancer risk in wild animals is anthropogenic food. Indeed, numerous animal species now inhabit nutritional environments distinct from their evolutionary past, a consequence of intentional or unintentional access to anthropogenic food sources, such as wildlife feeding or refuse sites (Giraudeau et al., 2018; Sepp et al., 2019). While there are many possible links between evolutionarily novel food and cancer (e.g., changed nutrient balance, consequences on immunity and inflammatory status, changed microbiome composition and oncogenic toxic contaminants; reviewed in Giraudeau et al., 2018), obesity in wild animals contributed by human food could be a starting point from the perspective of cancer in wild populations and evolutionary mismatch (Sepp et al., 2019). In humans and laboratory rodents, obesity has been associated with increased mortality risk from cancer, increased tumour aggressiveness, decreased response to treatment and a higher rate of cancer recurrence (Allott & Hursting, 2015; Haslam & James, 2005). Surprisingly, the link between obesity and cancer in pets is not well studied (Romano et al., 2016). Over the past several decades, body weights have risen among many groups of wild animals living in close contact with humans, and this phenomenon has been empirically linked to adverse health conditions (Beckmann & Lackey, 2008; Klimentidis et al., 2011; Maréchal et al., 2016; Schulte-Hostedde et al., 2018). These results highlight that species and populations overconsuming human food (e.g., racoons, some primates, bears and foxes (Murray et al., 2016)), could be good model organisms for understanding the evolutionary vulnerability of wild animals to obesity, and the link between obesity and cancer as one of the potential costs (Sepp et al., 2019).

### 2.4 | Feral species

Most non-invasive methodologies for diagnosing cancer (e.g., CT, MRI, endoscopy and blood tests) are routinely used in domesticated animals and the methodologies are precisely adjusted to these species. There is however a lack of practice in applying these methodologies in wild animals, which hinders the progress in understanding cancer in wild organisms. This is problematic, since understanding the ecology of cancer (e.g., life history trade-offs and role of environmental factors) could be best achieved by studying wild populations. A solution to this problem could be to study the ecology of cancer in feral animals, which have undergone the process of domestication but have then returned to the wild (Pierotti & Fogg, 2017). The available knowledge on the physiology of these animals, along with the established diagnostic tools routinely used in their domestic counterparts can aid the establishment of fascinating study systems. Feral populations of species such as dogs (*Canis lupus familiaris*), cats (*Felis catus*), sheep (*Ovies aries*) and horses (*Equus ferus caballus*) thus offer unique opportunities to study cancer under natural environmental conditions, by benefiting from easily applicable, state-of-the-art veterinary tools (Pesavento et al., 2018). Importantly, cancer is a major source of mortality in some feral animal populations. For example, CTVT disease is a major cause of mortality in feral dogs, especially in urbanized tropical and subtropical areas (Ganguly et al., 2016). Studying these feral-afflicted diseases has already contributed to knowledge about the evolution of cancer and cancer defences (Ujvari et al., 2018), and will likely continue to provide key insight into cancer biology in the future. For instance, studies comparing domestic and feral populations have already highlighted an effect of reproductive management on cancer prevalence. Specifically, domestic dogs and cats show a higher frequency of aggressive mammary tumours compared with their feral counterparts. Such phenomena are believed to be at least partly caused by chemical or physical breeding prevention in pets, resulting in their exposure to unnatural cycles of reproductive hormones (Munson & Moresco, 2007).

Pet and feral animals are exposed to contrasting environments, in respect to the consumption of processed foods, exposure to pollutants or artificial light at night (Sepp et al., 2019). Thus, exploring cancer risk in these animals could not only greatly aid our insight into cancer biology in general, but also improve our understanding of the role of environmental factors in shaping cancer risk (Giraudeau et al., 2018). Moreover, feral species (e.g., dogs, cats and pigeons) often inhabit urbanized areas, feed in refuse sites and are highly exposed to anthropogenic effects, allowing for the exploration of the oncogenic effects of these factors on cancer risk. Such a study was performed for instance in relation to the Kuwait oil fires, highlighting little or no long-term effect of inhaling smoke-contaminated air on the number and the severity of histopathological lesions in feral cats (Moeller et al., 1994). Moreover, studying multiple feral and pet species within the same environment could aid the identification of clusters of cancer cases, these being often difficult to identify in single-species setups (even in humans) due to confounding effects. Such cases are paramount in identifying environmental sources of carcinogenesis.

Taken together, studies using readily available methodologies (developed for domestic animals) in feral animal populations could be an important approach in the field of comparative oncology. Such studies could be highly informative, but would still represent an interim stage in the final goal of understanding wildlife cancer.

### 2.5 | Transmissible cancers

Transmissible cancers are among the most extensively studied neoplastic diseases in the wild. The pathogens are rogue, malignant cell lines that have derived and deviated directly from the host or from a closely related species, and they have acquired the capacity to spread among individuals and, in certain cases, among species. Currently, fourteen transmissible cancers (one in dogs, two in Tasmanian devils and eleven in distinct bivalve species) have been recorded in the wild, but their abundance has most likely been underestimated (Dujon et al., 2020; Ujvari et al., 2016a).

While transmissible cancers may appear infrequent, it is crucial not to underestimate their ecological and evolutionary ramifications. The narrative of the Tasmanian devils serves as a cautionary tale, where a clonal cell line spreading as an allograft is driving the once-abundant species to the brink of extinction (Hamede et al., 2020; Hawkins et al., 2006). Moreover, a novel, independent transmissible cancer lineage has recently emerged within the same species (Pye et al., 2016). The currently known transmissible cancers can provide guidelines to predict the species, ecosystems and environmental conditions, in which contagious cancer cell lines may emerge and prosper. For instance, the ever-increasing level of pollution threatening oceans globally could contribute to the emergence of contagious cell lines (Odintsova, 2020). Indeed, pollutants have been known to cause an increase in oxidative stress as well as damage to DNA, membrane lipids and proteins in marine species (see for instance Lerebours et al., 2013; Rhee et al., 2013). Of note, this environmental threat has already been linked to aquatic transmissible cancers. Specifically, disseminated neoplasia in bivalves appears at its highest frequency in polluted areas, often associated with high levels of heavy metals, PCBs and PAHs (Carballal et al., 2015). Further threats to aquatic ecosystems include increasing water temperatures, hypoxia, ocean acidification and overexploitation. Hypoxia and ocean acidification can impact physiological activities, such as metabolism and feeding performance of marine organisms (Wu, 2002) and could present a significant stressor initiating cancer development and progression. In addition, disseminated neoplasia cells can tolerate and thrive in low-pH (e.g., pH of 4.0, Sunila & Farley, 1989) and hypoxic environments, and thus, climate change may accelerate the emergence and support the persistence of cancer cell lines. The easy transmission route (i.e., water currents) and the ability of disseminated neoplasia cells to survive under various conditions (Sunila & Farley, 1989) identify aquatic environments as potential hotspots for transmissible cancers.

Cumulative effects of anthropogenic and environmental stressors can contribute to the collapse of local populations, leading to a loss of genetic diversity, which is another potential driver of cancer emergence (including transmissible cancers) (Belov, 2012; Ujvari et al., 2018). Indeed, the low genetic diversity of Tasmanian devils is currently thought to be a key source of their elevated cancer risk and their vulnerability to transmissible cancers (Belov, 2012; Stammnitz et al., 2018). Overall, perturbations to natural ecosystems with significant, anthropogenic impacts (e.g., pollution, UV exposure and climate change) in combination with habitat loss, subsequent local population collapses, and loss of genetic diversity can all contribute to an elevated risk of cancer in wild organisms. These, in combination with easy transmission routes (e.g., aquatic environments), could generate ideal conditions for the emergence of transmissible cancers. Current knowledge of these factors and of the diversity of transmissible neoplasia in bivalves, due to their life histories, reproductive tactics, immune system and easy transmission routes, highlights molluscs in aquatic ecosystems may be the most likely candidates to support the emergence of contagious cancer cell lines.

## 3 | METHODS TO STUDY WILDLIFE CANCER

In this section, we provide an overview of already available methods and tools that would be desired to be developed for the study of wildlife cancer.

### 3.1 | Longitudinal studies and wildlife health surveillance programmes

Longitudinal follow-ups of vertebrate populations with large sample sizes have drastically increased since the late 1960s, now spanning a wide spectrum of species with diverse life history strategies (Clutton-Brock & Sheldon, 2010). These monitoring programmes generally involve: (1) the standardized monitoring of known-age individuals, often for decades (even in short-lived individuals), which enables to precisely estimate demographic parameters such as age-specific mortality or reproductive success, and (2) the collection of local biotic and abiotic environmental features. Many non-invasive phenotypic traits (e.g., body mass and size, and external parasite infection) are routinely collected at each capture, thus providing fine-scale information related to the health and condition of the individuals (Cheynel et al., 2017; Sheldon et al., 2022). Interestingly, during long-term monitoring programmes, blood samples are often also collected. Such biological samples could be used as a robust tool to assess health status at each capture, even retrospectively. Long-term studies can also allow to study the age-specific physiology and disease risk, as well as the change in such processes according to changing environmental conditions (Sheldon et al., 2022). We also suggest the use of such biological samples (especially repeated samples of individuals) for ecotoxicological assessments, allowing to estimate lifetime ecotoxicological exposure of individuals, and to explore how this influences cancer risk (Reinke et al., 2019). Moreover, once diagnostic tools for wildlife cancer are established (see Section 2.5 below), investigating cancer dynamics in such populations will offer unique possibilities to understand how life history traits (e.g., growth and reproductive allocation) interact with environmental conditions (e.g., pollutants and pathogens) in shaping age-specific risk of cancer and how cancer ultimately affects age-specific mortality patterns (Lemaître et al., 2020). By applying standardized protocols in multiple longitudinal studies with diverse environments, an epidemiology-based approach can also be adopted to compare cancer rates between populations and species with and without specific exposures (e.g., comparing histological lesions in feral cats exposed or not to oil fires, Moeller et al., 1994). This approach is crucial for gaining general insights into the causes of cancer in wildlife.

In addition, wildlife health surveillance programmes have been established in many countries over the last decades with the aim to detect the emergence of diseases and predict potential zoonotic disease occurrences. Within these programmes, every year, tens of thousands of wild animals are examined or necropsied by trained pathologists (Kuiken et al., 2011). However, the resulting databases are so far rarely used to assess, especially with a decent sample size, cancer prevalence in a given species (but see Pewsner et al., 2017), nor to compare prevalence among species in a given habitat, abiotic or biotic environments. Such restriction may be partly due to a problem with database architecture. Indeed, these wildlife programmes are not typically organized in a manner that would allow data sharing (e.g., academic competition, career advancement goals and institutional policies) and standardization (e.g., standardized methodology, data structure or funding coverage that had been used consistently for decades are difficult to change). Moreover, there is a current need to establish common and standardized procedures to select organs for histological analyses for every animal that goes through a necropsy as part of these programmes, even for individuals with no suspicion of neoplasia. For instance, we urge wildlife services to inspect the following organs: liver, kidneys, lungs, mammary glands, lymph nodes, reproductive organs, spleen and the brain, and to preserve tissues for subsequent histological analyses whenever possible. Involving these programmes in the research on wildlife cancers would drastically increase our knowledge on cancer prevalence in wild populations with associated financial costs (beyond logistics and data sharing) given that many programmes are already in place, especially those exploring the source of mortality in wild animal carcasses.

### 3.2 | Data from zoos and aquariums

In the oncology context, the controlled environments of zoos and aquariums are especially relevant to understand causal factors for different types of cancer. However, it is important to keep in mind that the study of animals under human care in epidemiological and oncology studies has some limitations to the generalizability of the findings for wildlife due to the artificial environment and intensive management they are subjected to. Managed environments strip animals of natural conditions, including both abiotic and biotic factors. For instance, husbandry practices target the reduction in parasites, pathogens, harsh environments, predators and intra-sexual agonistic interactions. Consequently, some mortality factors present in the wild are eliminated under captive conditions, while animal physiology might be altered as well (Davey, 2007). Captivity also exposes animals to artificial stressors, such as changes in diet, lighting, reduced mobility, altered reproduction (e.g., suppressed reproduction in captivity, hormonal treatments and contraception), and the presence of caretakers and visitors (Morgan & Tromborg, 2007). While these factors may induce changes in diverse aspects of the animals’ biology compared with wild individuals, little is known concerning the susceptibility of each species or on how each aspect of physiology is affected (Davey, 2007; Morgan & Tromborg, 2007). It is thus imperative to consider these limitations when treating and interpreting data from animals under human care, irrespective of the characters of interest, in order to harness its great research potential (Conde et al., 2019).

Zoos and aquariums share standardized data across more than 21,000 species through Species360 (https://species360.org/), a non-profit membership-driven organization that manages and develops the Zoological Information Management Systems (ZIMS). Data on animal husbandry and health are shared across 1245 zoos and aquariums worldwide, enabling evidence-based management decisions to improve animal care. To date, ZIMS hosts 10 million husbandry and medical records for living animals and 800 million records for historical animals, some dating back to the late 1800s. These records cover all major taxonomic groups with diverse life history strategies, including one in seven threatened species of terrestrial vertebrates assessed by the International Union for Conservation of Nature (IUCN) Red List (Conde et al., 2011). Furthermore, ZIMS data contain sufficient sample sizes to develop analytics for at least 10% of all described extant birds and mammals with birth and death records, of which many have been subject to regular monitoring and medical scrutiny. Another database assessing cancer prevalence across species is the Exotic Species Cancer Research Alliance (ESCRA; www.escra.org). ESCRA is a global database that is working on cases of cancer from zoos and aquariums but also from wildlife and exotic pets to determine cancer prevalence, treatments and to start to gain an understanding of factors affecting survival.

The diversity and accessibility of captive animals, as well as the thoroughness of their medical records, provide an excellent basis for the identification of species with low cancer rates and potentially unique anticancer adaptations (Boddy et al., 2020). These species are of key importance to provide insights into the natural mechanisms of cancer resistance (Abegglen et al., 2015; Tollis et al., 2017). For example, traits that likely contribute to very low cancer rates include duplications of the TP53 tumour-suppressor gene in elephants, over-production of high molecular mass hyaluronan in the naked mole rats, interferon-mediated concerted cell death in the blind mole rats, and reduced growth hormone–insulin-like growth factor-1 signalling and microRNA changes in bats (Seluanov et al., 2018). The potential for developing anticancer therapies, especially non-toxic to the host organism, based on lineage- or species-specific mechanisms, is very high. The data in ZIMS and ESCRA therefore provide an unprecedented window of opportunity to pursue identifying species and mechanisms of interest (Vincze et al., 2022).

Contrary to most studies in the wild, individuals under human care are generally of known age, sex and pedigree, and in many cases with well-documented reproductive and medical histories, including detailed reports on anaesthesia, necropsies, management practices and reference intervals (e.g., haematology, toxicology, chemistry fluid analyses, serology-immunology, endocrinology and reproduction). Similar data resolution in the wild can only be achieved in populations subject to long-term monitoring. Nonetheless, following individuals in the wild is resource and work-intensive, while resulting data often suffer from data gaps (e.g., cause of death is rarely known). Moreover, the difficulty of performing such studies increases with species lifespan, rarity and inconspicuousness, resulting in biases in taxonomic sampling. Individual information is however important in identifying age-specific mortality risk factors and sex differences in pathological disorders (Lemaître et al., 2020) and in identifying reproductive or pathological associates of diseases (e.g., viruses). Additionally, known pedigrees allow the identification of hereditary pathologies and the determination of genetic risk factors for certain cancers, giving way to family-based genetic linkage studies (Easton et al., 1993).

Finally, carcasses of wild animals for research necropsies present high levels of autolysis or are rarely recovered, except those subject to accidents (e.g., roadkill or electric shock). Moreover, it can be proposed that individuals subject to physiological decline (e.g., due to an early stage of cancer) may be generally at a higher risk of predation and more likely to succumb to otherwise benign infections, biasing cancer mortality estimates. In contrast, in zoos and aquariums, most deceased animals are recovered by caretakers and necropsies are routinely performed, helping the identification of neoplastic cell growths, species and organs of interest (including co-morbidity information), providing plenty of material for histological scrutiny (Iacobuzio-Donahue et al., 2019).

### 3.3 | The need to generalize histological analyses

Histopathology is used to distinguish neoplastic diseases from inflammatory and/or infectious processes that may macroscopically manifest similarly. Histology is the hallmark tool to study the nature of the cancerous cell and determine its origin, growth potential, aggressiveness and ability to spread via lymphatic and blood vessels. This may assist the clinicians in the prognosis of wild animals in a captive setting (i.e., through biopsies of animals showing signs of illness) but also during necropsies performed through wildlife health surveillance programmes. In addition, when target tissues of early neoplastic transformations are systematically sampled (e.g., especially in organs prone to cancer development, see Section 2.1), this methodology can also be used to detect preneoplastic lesions. Applying histology in the search for cancer in wildlife is a particularly promising tool to increase general knowledge about cancer in these species. In this context and for the sake of comparability, the use of standardized tissue collection, sample processing and analysis are encouraged.

Conventional histology involves collecting samples from all organs and any visibly abnormal adjacent tissue. A representative sample includes a section of the lesion and the junction with surrounding normal tissues, fixed in neutral buffered formalin. It is recommended that samples not be stored in neutral buffered formalin for more than 24 h before paraffin embedding to ensure optimal antigen retrieval for ancillary testing (Guerrero et al., 1997; Ramos-Vara et al., 2008; Werner et al., 2000). Tissues are then sectioned and stained with haematoxylin and eosin for optical microscopic examination. Retaining samples after histological examination in the form of formalin blocks is a valuable resource for retrospective studies and is strongly recommended. In cases when standard histologic examination is not sufficient to determine the tissue of origin, the same tissue samples may undergo analysis with specific immunohistochemical (IHC) stains to aid in identifying the nature of the neoplasm. It is important to note that while these specific tests have been developed for human cancer, several have also been successfully applied to animal cancer, including wildlife cases. However, it should be emphasized that markers (protein targets and antibodies required for detection) that work for IHC in some species may not work in others, and successful application also depends on tissue and tumour types (Hammer et al., 2007; McAloose & Newton, 2009). For instance, antibodies developed for human proteins might not recognize the same protein in another taxa due to sequence differences. Additionally, PCR, immunofluorescent methods, tissue microarrays or electron microscopy of tumours are alternative techniques that have been successfully employed for robust diagnosis and prognosis evaluation (PCR: detection of the Otarine herpesvirus-1, King et al., 2002; immunofluorescent approach: association of the dubbed polyomavirus with racoon brain tumours’ presence, Dela Cruz et al., 2013; tissue microarray: malignant lymphoma diagnosis in a manatee, Hammer et al., 2007; electron microscopy: description of a herpesvirus-like virus in green turtles with Fibropapillomatosis, Aguirre & Spraker, 1996).

### 3.4 | Liquid biopsies

During the last decade, liquid biopsy techniques (including the measurement of circulating tumour cells [CTCs], exosomes or circulating cell-free DNA) have provided new insights into the biology of metastasis, with important implications for the clinical management of cancer patients using precision medicine (Cortés-Hernández et al., 2020). CTCs are cells shed by primary tumours into the vascular system. They are rare events in the bloodstream (e.g., 1–10 CTCs *per* 7.5 mL) and thus need to be concentrated, enumerated and isolated. As CTCs are surrounded by millions of normal leukocytes, different technologies are needed to concentrate them. These enrichment methods rely on different properties of CTCs that distinguish them from immune cells, including biological properties (e.g., targeting membrane proteins such as the epithelial cell adhesion molecule) and physical properties (e.g., size, density, electric charges and deformability). Following this enrichment step, the CTC fraction may still contain a substantial number of leukocytes; thus, CTCs need to be identified at the individual level. Immunological detection is the predominant approach used for CTC detection using antibodies directed against membrane and intra-cytoplasmic antigens (Pantel & Alix-Panabières, 2019). The only FDA-approved technology on the market is the CellSearch^®^ system, and most current CTC assays use the same identification steps (but see Habli et al., 2020 for alternative technologies). Cells stained with fluorescently labelled antibodies to epithelial cytokeratins are visualized through fluorescence microscopy and used as markers of CTCs, whereas staining of CD45 is used for leukocyte exclusion.

Marconato et al. (2019) enumerated, for the first time, CTCs in canine metastatic mammary carcinoma with the automated CellSearch^®^ platform. They detected at least one CTC *per* 7.5 mL of peripheral blood in 12 of 27 samples (44.4%), while no CTCs were found in healthy, negative control dogs (*N* = 5). The presence of CTCs was predictive of short survival in the canine cohort. These observations identified the first actionable marker in veterinary oncology to guide the treatment of canine metastatic mammary carcinoma (Chmielewska et al., 2013). Moreover, Wright et al. (2019) reported flow cytometry detection of circulating osteosarcoma cells in dogs with a strong increase within 4 weeks before overt metastases or death. These preliminary observations suggest that CTCs are frequent in canine osteosarcoma and that an increase in CTC frequency may foretell clinical deterioration.

The detection of CTCs is a methodology that still requires additional developmental steps before being considered as a standard tool in human or veterinary medicine. It could also constitute a very promising tool to diagnose cancers in blood samples collected in wild vertebrates. In this line, an immediate goal will be to evaluate whether current technologies for CTC analyses in humans can be applied to non-human species (such as the CellSearch^®^ system in dogs). Antibodies available for CTC detection in humans will also need to be tested in domestic and wild animals, or new animal-specific antibodies for CTCs will need to be developed. Finally, the current version of this test requires at least 7.5 mL for CTC counting, which can be problematic to obtain from small taxa. Indeed, this represents a significant volume of blood considering that national ethical statements mainly postulate not to exceed 1%—1 mL *per* 100 g—of the animal’s body weight. Therefore, the refinement of such methodology to reduce the blood quantity required for the analysis would be conducted.

In medicine, genomic analyses of liquid biopsies targeting circulating tumour DNA (either cell-free or not) have been leveraged for the early detection of cancers. These assays can either rely on sensitive detection of cancer-associated mutations (Chaudhuri et al., 2017; Merker et al., 2018) or on chromatin/epigenetic patterns indicative of the cancer transcriptional phenotype (Cristiano et al., 2019; Liu et al., 2020). While these assays can have high specificity and sensitivity, particularly for later stage cancers, the costs associated with false positive results have thus far impeded their widespread use. In contrast, liquid biopsies, as methods to genotype already diagnosed cancers, are widely used clinically (Crowley et al., 2013).

In total, these mutation-detection assays could allow for sensitive discovery of cancers in wild animals with a unique blood sample (a few millilitres would suffice). However, in addition to the methodological constraints/costs associated with such collection, some hurdles have to be reported. First, these assays require the capture and sequencing of specific regions of the genome and thus may be limited to species with sequenced genomes or their close relatives. Second, some information on the mutational profiles of cancers in these species would be required so that appropriate regions could be targeted. Nonetheless, we do know that common genes contribute to cancers across mammalian species (such as in TP53), and thus, initial attempts could focus on the ‘usual suspects’. In addition, as more tumours from wild animals are genomically profiled, panels could be created for these species that could allow rapid screening of many individuals. Such panels could be particularly useful for species with high cancer incidence.

For the DFTD, where the full genomes of both transmissible cancers are available, panels could be designed to allow early detection of these cancers in devils. Such early detection could improve outcomes for interventions (as early detection clearly does for humans) and could also provide insight into DFTD–host interactions. For example, such screening may reveal the frequency to which devils are able to reject an early infestation with the cancer, with subsequent capture of the same individuals revealing no disease. While further development would be required, early detection and characterization of the mutational landscapes of cancers in wild animals could improve our understanding of cancer prevalence, host responses and potential interventions.

### 3.5 | Metabolomics

Cancer is characterized by abnormal metabolism, including consequences of hypoxia such as increased glycolysis, decreased oxidative phosphorylation, and specific impairments of protein and lipid synthesis, but also adaptations that support the unusually high rates of cell growth and proliferation found in tumours (DeBerardinis, 2008). As technological improvements (mainly based on mass spectrometry and nuclear magnetic resonance spectrometry) increase the feasibility of studying tumour metabolism, an increasing number of studies have reported the molecular connections between cancerous processes and cell metabolism (Kaushik & DeBerardinis, 2018). The application of metabolomics (i.e., the scientific study of comprehensive sets of metabolites), present in biological samples, has shown great potential in disease diagnosis, prognosis and patient stratification in most types of tumours in humans (Wang et al., 2016). Since various biofluids (e.g., blood, urine, faeces and other biological matrices) can be used for validating metabolic biomarkers noninvasively in human cancer patients (Serkova & Glunde, 2009), this method is appealing for application in wild animal research. However, as the metabolome is highly influenced by factors such as genetics, the environment and genotype-by-environment interactions, metabolomics is still in its early stages and has many unsolved challenges (Peluc et al., 2012). This is especially true for wild animals, where additional difficulties associated with limited sample availability, sampling bias, conditions for sample collection, nutritional status and seasonal variation are known to affect the results of metabolomics analysis (Bundy et al., 2009; Griffin & Kauppinen, 2007; Karu et al., 2016; Miller, 2007). Before metabolomics can be applied as a minimally invasive method for assessing cancer prevalence in the field, considerable effort must be dedicated to validating this method on model species and prevalent cancer types. Since the metabolic pathways related to cancer are often evolutionarily conserved across eukaryotes, studies in humans and laboratory models still offer a good starting point. For example, in a mouse model of pancreatic ductal adenocarcinoma, metabolomic analysis of serum distinguished animals with early- or late-stage lesions from respective controls with more than 80% accuracy (LaConti et al., 2015). The first step in applying metabolomics research to studying cancer in wild animals would therefore be to select a model species with a known high prevalence of some type of cancer, which has also been studied with a metabolomic approach in humans. The use of this method should then be validated on individuals of different age, body size, sex and disease stage to account for phenotype–environment interaction effects on metabolomics, potentially also using different tissue types such as blood or urine. As a first example of applying metabolomics to studying wildlife cancer, a study on Tasmanian devils provided a relevant set of biomarkers for diagnosing DFTD, many of which were useful in the early stages of the disease (Karu et al., 2016).

### 3.6 | Cancer comparative genomics and anticancer defences

Another approach to studying cancer in wildlife is through comparative genomics and transcriptomics. Comparative genomics and transcriptomics can identify anticancer defence mechanisms and reveal an organism’s underlying genetic diversity. Cancer comparative genomics can reveal the evolution of anticancer mechanisms (as reviewed in Tollis et al., 2017) through gene expansion or adaptive evolution of tumour-suppressor genes. Cancer comparative genomics has focused on species with slow life histories, such as species with high body mass (such as elephants, Abegglen et al., 2015; Sulak et al., 2016 and whales, Keane et al., 2015; Tollis et al., 2019) or long lifespans (such as naked mole rats, Tian et al., 2013; blind mole rats, Manov et al., 2013 and Brandt’s bats, Zhang et al., 2013).

Based on our limited studies of cancer comparative genomics, our current understanding of anticancer defence mechanisms in large or long-lived species includes more efficient DNA repair mechanisms and a higher sensitivity to DNA damage, such as cell contact inhibition and apoptosis (Abegglen et al., 2015; Seluanov et al., 2018; Sulak et al., 2016). Careful functional studies are still needed to tease apart the mechanisms underlying these cancer defences. More efficient DNA repair or a higher sensitivity to DNA damage may protect a species from neoplastic growth and progression and these mechanisms are important contributions to the somatic maintenance of individuals. However, according to life history theory, efforts in somatic maintenance, including DNA repair, cell cycle control and immune function, are costly and subject to trade-offs (Boddy et al., 2015). Quantifying the costs of cancer defences and determining the specific trade-offs will be an important new direction in comparative oncology. Initially, one could make predictions rooted in life history theory about allocation to anticancer defences as well as their dependence on the ecology, reproductive biology or energy expenditure of individuals and species. For instance, we can anticipate that anticancer mechanisms are more likely to evolve in taxa that have a late and slow rate of reproduction, or taxa that benefit from longevity over fast reproduction.

As stated earlier, environmental change can lead to evolutionary mismatches between adaptations to the expected (historical) and current environment of organisms. Species affected by habitat loss and fragmentation will likely go through a population bottleneck and reduction in genetic diversity. With small population sizes, rare (and sometimes deleterious) alleles can rise to high frequencies and become overrepresented in a population. Natural selection, which weeds out these deleterious alleles in large populations, is less efficient in small populations, which are usually dominated by genetic drift (Ohta, 1973). Using these general principles of population genetics, we can predict that animals with a substantial population bottleneck may experience higher rates of cancer through random drift and fixation of deleterious alleles, such as mutations in tumour-suppressor genes, as well as consequent loss of genetic diversity (i.e., heterozygosity) (Belov, 2011). Loss of genetic diversity can also lead to decreased immunosurveillance, which may lead to more viral infections and less surveillance of cancer cells within the host, both of which can contribute to cancer susceptibility (Belov, 2011, 2012). Measuring the genetic diversity of a vulnerable population, including specific alleles present at the major histocompatibility complex locus, can be an important indicator of health (Frankham, 2005; Grogan et al., 2017; Ujvari & Belov, 2011) and may be a key target for wildlife cancer observation programmes.

Additionally, while monitoring populations that have recently undergone genetic bottlenecks will be an important method for detecting wildlife cancer, wildlife cancer programmes may need to prioritize species that are classified on the slow end of the life history continuum. Species with slow life histories might be most vulnerable to cancer, as slower pace of life can delay the recovery of genetic diversity. Measuring genetic diversity will require blood, tissue (or potentially faeces) collection to isolate and genotype the DNA of multiple individuals in the same population. The collection of some of these samples is considered invasive and may be difficult to perform for endangered species. Thus, it is critical for wildlife cancer biologists to work in line with global and/or local animal conservation programmes to help facilitate successful collection. In such cases, interdisciplinary teams of biologists, veterinary pathologists, oncologists, conservationists and ecologists will be needed to develop successful research programmes on wildlife cancer.

## 4 | CONCLUSIONS AND FUTURE PERSPECTIVES

One of our most powerful tools for understanding how natural selection has shaped biology is through comparisons of different organisms and the identification of convergent evolution. This approach has been almost entirely lacking in the study of cancer. The little comparative oncology data that have been published are almost exclusively from specific species and from animals in captivity. What solutions has nature found for preventing different types of cancer? What makes an organism vulnerable to a type of cancer? What proportion of cancers in the wild are caused by viruses and which viruses cause them? Answering these questions will undoubtedly provide a better understanding of cancer in humans and animals alike. Animals under human care in zoos and aquariums can provide rich data on individual cases, but caution is needed due to the artificial environment. Thus, understanding the evolution of cancer defences will require the collection of data from animals in the wild.

We have outlined methods to identify species for study that are most likely to provide valuable insights. These include species that are outliers in cancer-relevant characters, such as animals that have a much longer or shorter lifespan than would be expected given their body size. Also, we might make progress by examining species whose habitats have recently changed (e.g., urban and/or polluted environments) and so may be in an evolutionary mismatch with their environments, much like humans. Similarly, animals that have undergone recent and rapid evolutionary change may have cancer vulnerabilities because the selective effects of cancer may have been overwhelmed by selection for the rapidly changing trait.

There are a number of challenges in collecting cancer data from animals in the wild. It is our hope that highlighting the value of these data will help drive efforts to overcome those challenges. These include the logistics of surveying wild animal populations, detecting cancers that are not externally visible, collecting biopsies from tumours and recovering carcasses of deceased animals before they decay or are consumed. We have suggested potential methods to study cancers in wildlife, including longitudinal studies of wild populations, teaming up with wildlife management efforts and using liquid biopsies to detect cancers. Studies of cancer prevalence can be paired with comparative genomic analyses to identify potential molecular mechanisms of cancer defences, as has been shown in elephants (Abegglen et al., 2015), whales (Tollis et al., 2019) and mammals (Tollis et al., 2020) more generally. These observations may then be tested in follow-up, controlled experiments.

Ultimately, over millions of years of evolution, species have been exposed to cancer and evolved mechanisms to suppress it. Understanding these mechanisms across the tree of life and their interactions with the environment will contribute to the One Health approach (Box 2) and thus will allow us to discover new ways to guide new therapeutic strategies.

## BOX 1 A note of caution about wildlife oncology

Studies of clinical and veterinary oncology have accustomed researchers to clearly distinguish between benign and malignant tumours, as well as cancers of different organs (e.g., cervical *vs* breast cancer) and of different genetic or molecular features (e.g., Hodgkin *vs* non-Hodgkin lymphoma). However, such distinctions remain highly challenging when exploring neoplasia in non-model organisms. The reason for this is manifold. First and foremost, data are currently extremely scarce on the occurrence of tumours, of any type, in non-model animals, especially from free-ranging, wild animal taxa. Second, recently compiled databases using data from zoo animals provided key insight in the frequency of tumours in wild animals under human care. Nonetheless, these databases and even the underlying diagnostic report often fail to provide information on the nature (benign *vs* malignant), site (i.e., organ) of the tumours or on the results of the histological analyses (if performed). Consequently, finding data on the risk of benign and malignant tumours from wildlife is highly difficult. Moreover, data on the organ or type of the tumour/cancer are virtually non-existent in currently available databases and resources.

While the lack of information on tumour malignancy, site or type in non-model organisms is partly problematic, this should not discourage scientists from exploring the risk of neoplasia *per se*, as a proxy for cancer risk across species for multiple reasons. First, deep-level genomic sequencing has recently provided evidence that certain genetic alterations traditionally listed as hallmarks of specific cancers, can be also present in benign and pre-malignant tumours, often in much higher frequencies than in malignant neoplasms (Kato et al., 2016). This observation suggests that the distinction between benign and malignant tumours is not always straightforward and the underlying molecular mechanisms can be shared. Consequently, studying the frequency of neoplasms, without distinction of malignant and non-malignant tumours might be a highly informative tool to understand oncogenesis, especially in a comparative framework. This notion is further supported by a recent preprint amassing information on necropsy reports of zoo vertebrates, highlighting the strong positive correlation between the frequency of malignant and benign tumours across species (Compton et al., 2023). This suggests that resistance to cancer implies resistance to benign tumours as well and that studies without distinction of cancer types remain highly valuable for understanding tumour biology, even if interpretations and conclusions based on these should be approached with caution. Nonetheless, future work should aim to gather precise information on the type and location of tumours for easier analysis, interpretation, generalization and distinction of underlying processes.

## BOX 2 The ‘One Health’ perspective, wildlife and human cancer

Since its creation in 2004, the ‘One World, One Health’ concept (i.e., the health of humans being closely linked to the health of animals and our shared environment), has attracted considerable attention. The relevance of this approach is becoming increasingly evident with the accelerated emergence rate of zoonoses over the last few decades (e.g., Ebola, SARS, MERS and the current COVID-19 pandemic) (Peyre et al., 2021). Although typically presented as holistic, the One Health approach has often remained focused on zoonotic diseases. Recent attempts to broaden the scope led to the inclusion of other fields, such as ecotoxicology, antimicrobial resistance and health in urban environments (Destoumieux-Garzón et al., 2018). It has recently been recognized that including cancer as a stake in the One Health concept is urgently needed as well (Dujon et al., 2021). First, various links exist between oncogenic processes and the three main components of the One Health approach. For instance, numerous human activities are considered to be oncogenic for wildlife species and exacerbate the dynamics of oncogenic processes in animals (e.g., see Giraudeau et al., 2018; Sepp et al., 2019). In parallel, oncogenic processes are known to generate a range of immunosuppressive disorders, sometimes during the early stages of oncogenesis (Pollock & Roth, 1989). Based on studies in human and captive animals, it is predictable that oncogenic processes also cause immunosuppressive disorders in wild animals. Therefore, the evolutionary mismatch between novel cancer risks in human-driven ecosystems and maladapted cancer defence levels in animals is expected to indirectly result in heightened pathogen dynamics in wildlife/ecosystems. The extent to which these processes lead to the transfer of increased pathogen communities to human societies/populations remains to be answered. Pollution-induced oncogenesis could also amplify these transfers if it concomitantly promotes a decline in biodiversity, hence reducing the protection conferred by the dilution effect (Civitello et al., 2015).

A second reason to give stronger importance to cancerous pathologies in the One Health approach is the fact that cancers can themselves become transmissible in certain contexts. At the moment, fourteen transmissible cancer lineages have been discovered (one in dogs, two with devastating effects in Tasmanian devils’ populations, and eleven in marine bivalves, Dujon, Gatenby, et al., 2020; Hammel et al., 2024), but this number is very likely underestimated (Ujvari et al., 2016b). Conditions for the emergence of transmissible cancers are still poorly understood, but this information is crucial: like other infectious diseases, transmissible cancers have the potential to further damage ecosystems and accelerate biodiversity loss (e.g., see Bramwell et al., 2021; Hollings et al., 2016). Finally, a third reason to consider cancer in the One Health approach lies in the scientific insights provided by studies exploring natural cancer defences in the animal kingdom. For example, contrary to theoretical expectations, there is no correlation between body size, longevity and cancer rates across species because of the various cancer resistance/tolerance mechanisms that have evolved in large and long-lived species (i.e., Peto’s Paradox, Nunney et al., 2015; Vincze et al., 2022). Scientists are increasingly able to decipher the underlying mechanisms in these cancer-resistant/tolerant species (Abegglen et al., 2015; Keane et al., 2015; Sulak et al., 2016). Another particularly interesting direction of comparative oncology is the study of animals that have lived in naturally polluted areas for eons (Vittecoq et al., 2018). The long-term exposure of a population to ecological contexts that exacerbate oncogenic processes should efficiently select individuals whose fitness is—by one way or another—less affected by cancer burden. From an applied perspective, studying these adaptations could also inspire nature-based solutions to prevent and/or to treat cancer by mimicking the processes allowing these species to prevent or limit malignant progression despite high levels of mutagenic substances. Thus, comparative oncology is the key to understanding cancer epidemiology, prevention and improved therapies for humans.
